# Spontaneous calcium activity in metanephric mesenchymal cells regulates branching morphogenesis in the embryonic kidney

**DOI:** 10.1096/fj.201802054R

**Published:** 2018-11-29

**Authors:** Jacopo M. Fontana, Georgiy R. Khodus, David Unnersjö-Jess, Hans Blom, Anita Aperia, Hjalmar Brismar

**Affiliations:** * Department of Applied Physics, Science for Life Laboratory, Kungliga Tekniska Högskolan (KTH) Royal Institute of Technology, Solna, Sweden;; † Department of Women’s and Children’s Health, Science for Life Laboratory, Karolinska Institutet, Solna, Sweden

**Keywords:** organogenesis, nephron, calcium imaging

## Abstract

The central role of calcium signaling during development of early vertebrates is well documented, but little is known about its role in mammalian embryogenesis. We have used immunofluorescence and time-lapse calcium imaging of cultured explanted embryonic rat kidneys to study the role of calcium signaling for branching morphogenesis. In mesenchymal cells, we recorded spontaneous calcium activity that was characterized by irregular calcium transients. The calcium signals were dependent on release of calcium from intracellular stores in the endoplasmic reticulum. Down-regulation of the calcium activity, both by blocking the sarco-endoplasmic reticulum Ca^2+^-ATPase and by chelating cytosolic calcium, resulted in retardation of branching morphogenesis and a reduced formation of primitive nephrons but had no effect on cell proliferation. We propose that spontaneous calcium activity contributes with a stochastic factor to the self-organizing process that controls branching morphogenesis, a major determinant of the ultimate number of nephrons in the kidney.—Fontana, J. M., Khodus, G. R., Unnersjö-Jess, D., Blom, H., Aperia, A., Brismar, H. Spontaneous calcium activity in metanephric mesenchymal cells regulates branching morphogenesis in the embryonic kidney.

Formation of the functional units of the human kidney, the nephrons, occurs during the second and third embryonic trimester and cannot be resumed later in life (1). Fetal malnutrition and other forms of adverse developmental programming can severely retard nephron formation. Low nephron endowment is a risk factor for progressive chronic kidney disease (2). Understanding nephron morphogenesis in greater detail could improve our possibilities to treat retarded kidney development.

Nephron formation is initiated by the reciprocal interaction between metanephric mesenchyme (MM) cells and ureteric bud (UB) cells and is mediated by paracrine morphogenic factors produced in MM that act on UB receptors and vice versa. This reciprocal interaction stimulates the outgrowth and branching of the UB cells into MM cells and the mesenchymal-epithelial transformation of MM cells into nephrons. The glomerulus, proximal tubule, loop of Henle, and distal tubule are all formed from transformed MM cells, and the collecting duct is in parallel formed from UB branches. This process of branching morphogenesis drives the formation of nephrons and is a major determinant of the ultimate number of nephrons in the kidney.

Branching morphogenesis is a fundamental development process in plants and animals and attracts the attention of experimental and theoretical scientists in different fields. In a series of theoretical studies, it has recently been proposed that morphogenesis in kidney, lung, and mammary glands can be described as a self-organized process (3) with stochastic components that are modified and controlled by signaling molecules (4–6). This description is supported by several studies of the embryonic kidney, where it has been demonstrated that genetically controlled and programmed events play a role for proper branching morphogenesis (7).

In the present study, we examined the role of spontaneous calcium signals for branching morphogenesis in the rat embryonic kidney. Calcium signals play a central role in embryo- and organogenesis. In a previous study on the role of apoptosis for adverse developmental programming of the kidney, we observed spontaneous calcium signals in explant kidneys from embryonic rats (8). Serum deprivation, which is used to mimic malnutrition, was found to down-regulate the spontaneous calcium activity and to retard nephron formation. In the current study, we have characterized this spontaneous calcium activity using kidneys derived from 14-d-old rat embryos (9). After having identified the endoplasmic reticulum (ER) as the major source of spontaneous calcium signals in the embryonic rat kidney, we examined how those signals contribute to regulation of branching morphogenesis during embryonic kidney development.

## MATERIALS AND METHODS

### Organ culture

The Stockholm North Ethical Evaluation Board approved all animal experiments. The studies were performed on kidneys from 14-d-old Sprague-Dawley rat embryos (d 0 of gestation coincided with the appearance of the vaginal plug in timed pregnant female rats), and Syt1 knockout mice (Syt1tm1Sud; The Jackson Laboratory, Bar Harbor, ME, USA). Kidneys were cultured as described by Sebinger *et al.* (10). Briefly, kidneys were cultured on 10-mm-diameter coverslips (1.5; Menzel, Kuchen, Germany) within a tissue culture insert (flexiPerm ConA; Sarstedt, Nümbrecht, Germany) with ∼85 μl of DMEM/F12 (Thermo Fisher Scientific, Stockholm, Sweden) supplemented with 10% fetal bovine serum (Thermo Fisher Scientific). The culture insert with coverslip was placed in a 35-mm-diameter culture dish, and 2 ml culture medium was added around the insert as a humidifying buffer. Kidneys were cultured 24 or 48 h at 37°C in an air atmosphere containing 5% CO_2_ and 90% humidity.

### Chemicals

Cyclopiazonic acid (CPA), EGTA, and nifedipine were purchased from MilliporeSigma Sweden AB (Stockholm, Sweden). Ryanodine was purchased from Tocris Bioscience (Bristol, United Kingdom). BTP2 was purchased from MilliporeSigma.

### Calcium imaging

Kidneys from 14-d-old embryonic rats (E14) were cultured for 1 or 2 [days *in vitro* (DIV)1 and DIV2]. Fluorescence imaging of spontaneous calcium activity was performed in explanted kidneys using the calcium indicator dye Oregon Green BAPTA-1 acetoxymethyl (AM). This dye, like most calcium indicators, is unable to cross lipid membranes and is therefore loaded into the sample in a cell-permeable AM ester form. Inside cells, esterase cleaves the AM groups, and the dye becomes trapped in the cell.

**Figure 1:**
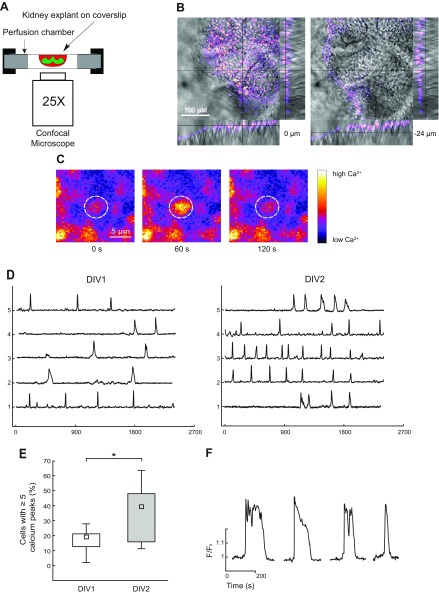
Recording of spontaneous calcium activity in the methanephric mesenchymal cell layer. *A*) Principal scheme of the setup. *B*) Orthogonal view of embryonic kidney at position 0 and −24 µm from the surface of the kidney explant. Recorded in transmitted (gray scale) and fluorescent light (pseudocolors) simultaneously. The signal from the fluorescent calcium dye Oregon Green BAPTA-1 AM is only detectable in the outer cell layers. *C*) Example of a single calcium spike in an MM cell. *D*) Forty minutes of recording of spontaneous calcium activity in 5 separate cells from kidneys at DIV1 and DIV2. Traces are presented as normalized fluorescence intensity (*F*/*F*_0_). *E*) Calcium activity increases from DIV1 to DIV2. Box plot of the relative number of cells that displayed multiple (5 or more) calcium peaks during the 40 min observation period. Statistical significance was calculated by 2-tailed, nonparametric *U* test (*P* < 0.05) for 11 kidneys in DIV1 and 13 DIV2 kidneys. *F*) Calcium traces taken from 4 randomly selected cells show the high variability in shape and duration of calcium spikes.

Kidney explants were loaded for 50–60 min with 5 µM Oregon Green BAPTA-1 acetoxymethyl ester and 0.04% pluronic acid F-127 (Thermo Fisher Scientific) at 37°C in a 5% CO_2_ air atmosphere. The calcium affinity of Oregon Green 488 BAPTA-1 AM is relatively high (*K*_d_ ∼170 nm), which can be advantageous for detecting small changes in Ca^2+^ near resting concentration levels (11). After the loading period, the coverslip was placed in a temperature-controlled perfusion chamber with constant fluid flow (FCS2; Bioptech, Butler, PA, USA), and temperature was maintained at 37°C. The chamber was mounted on a microscope (Axiovert LSM 510; Zeiss, Oberkochen, Germany) with a ×25 numerical aperture (NA) 0.8 water-immersion objective (Zeiss). For experimental setup design, see **Fig. 1*A***. Cells were imaged at 0.5 Hz using a 488 nm argon laser and an LP 505 long pass filter.

All experiments were performed in Krebs ringer buffer solution containing 100 mM NaCl 4 mM KCl, 20 mM Hepes, 25 mM NaHCO_3_, 1 mM CaCl_2_, 1.2 mM MgCl_2_, 1 mM NaH_2_PO_4_⋅H_2_O, and 10 mM D-glucose. In Ca^2+^-free solution, Ca^2+^ was substituted with 2 mM NaCl, and 250 μM EGTA was added. Solutions were titrated to pH 7.4 with NaOH. The osmolality of the extracellular solution, as measured with an osmometer (5500; Wescor, Salt Lake City, UT, USA), was 300–310 mmol/kg.

### Immunofluorescence

Kidney explants were fixed using −20°C methanol for 10 min and rinsed several times in PBS containing 0.1% Triton X-100 (PBST). Antibodies were then added to PBST buffer containing either 5% normal donkey serum or 5% bovine serum albumin, depending on the secondary antibody used, and incubated overnight at room temperature or 4°C. Samples were rinsed several times in PBST, and the secondary antibodies were then added to PBST containing either 5% normal donkey serum or 5% bovine serum albumin and incubated at room temperature for 4 h. Samples were then rinsed several times in PBST and mounted on microscope slides in Immu-Mount (Thermo Fisher Scientific) or in 80.2% (w/w) fructose solution containing 0.5% α-thioglycerol and left for 2 h at room temperature to allow for diffusion of mounting medium into the sample.

The antibodies used in this study were rabbit anti-WT1 (1:200, C-19; Santa Cruz Biotechnology, Dallas, TX, USA) and mouse anti–E-cadherin (1:500, 610182; BD Biosciences, San Jose, CA, USA). The specificity of each antibody described was confirmed either by Western blot analysis or preimmunization with a blocking peptide. The secondary antibodies used were goat anti-mouse Alexa 488, goat anti-rabbit Alexa 546, donkey anti-rabbit Alexa 568, and donkey anti-mouse Alexa 647 (all from Thermo Fisher Scientific).

All immunofluorescence images were obtained with a Zeiss LSM 780 or LSM 510 confocal system with either a ×20 NA 0.8 air objective, a ×25 NA 0.8 water-immersion objective, or a ×63 NA 1.4 oil-immersion objective (Zeiss).

### Proliferation and apoptosis

The apoptotic index was determined using the ApopTag Red *In Situ* Apoptosis Detection Kit (MilliporeSigma). Cell proliferation was detected and quantified using EdU incorporation assay with the Click-iT EdU Alexa Fluor 647 Imaging Kit (Thermo Fisher Scientific).

### Image analysis

Image analysis was performed using ImageJ (National Institutes of Health, Bethesda, MD, USA; *http://rsbweb.nih.gov/ij/*) and custom-made scripts in MatLab (MathWorks, Natick, MA, USA).

**Figure 2:**
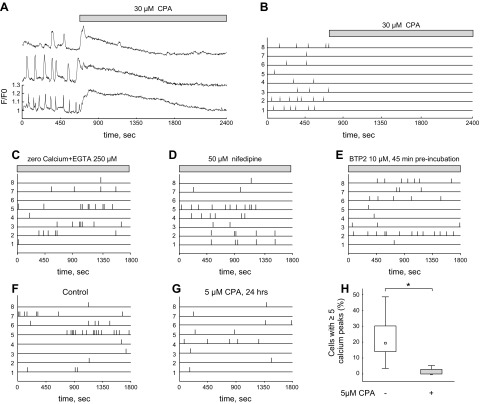
Calcium stores in the endoplasmic reticulum are the main source for spontaneous calcium activity in MM cells. *A*) Application of 30 µM CPA caused almost complete inhibition of calcium transients after an initial rise in intracellular calcium. *B*–*E*) Binary representation of spontaneous calcium transients from 8 randomly selected MM cells exposed to 30 µM CPA (*B*), calcium-free solution (250 µM EGTA) (*C*), 50 µM nifedipine (*D*), and 10 µM BTP-2 (*E*). *F*, *G*) Chronic application of 5 µM CPA from DIV1 to DIV2. *H*) Comparison of calcium activity in control and CPA-treated kidneys. Box plot represents the relative number of cells that displayed multiple (≥5) calcium spikes in 5 kidneys in both the control and CPA-treated groups. Statistical significance was calculated by 2-tailed, nonparametric *U* test (*P* < 0.05).

The calcium recordings were filtered for noise and normalized to baseline fluorescence using running median filter and wavelet transforms (Daubechies third order). A calcium spike was defined as a signal amplitude increase of >10% over baseline. Spikes were identified for all cells and plotted in a spikes/time diagram (**Fig. 2*B*–*G***).

Primitive glomeruli and UB tips were counted in the largest sagittal cross-section from each kidney. The number of glomeruli and ureter bud tips per maximal cross-section was taken as a reflection of total kidney primitive glomeruli and ureteric tip numbers (12). Data for each experimental group were normalized to the control for each separate culture preparation (kidneys in 10% FBS during the entire culture period).

### Statistical analysis

Calcium activity data are expressed as median, and differences were evaluated by a 2-tailed Mann-Whitney *U* test. For morphologic parameter counting, apoptosis detection and cell proliferation assay data are expressed as means ± sem. Differences between means were evaluated by 1-way ANOVA. Statistical significance was accepted at *P* < 0.05.

## RESULTS

### Metanephric mesenchyme cells exhibit spontaneous calcium activity

Because only the first cell layer of a sample is permeable to the calcium indicator, we used in the preparations of explanted and cultured kidneys (see Materials and Methods). The dye was, as expected, primarily loaded in the outer kidney cell layer. This layer consists to a large extent of MM cells, which surround and condense around the developing UB (Fig. 1*B*). Most cells displayed spontaneous calcium activity (Fig. 1*C*, *D*). The calcium signals appeared to be stochastic and were characterized as transients with irregular shape, amplitude, and duration (Fig. 1*D*, *F*). The number of actively signaling cells (defined as ≥5 transients during a 40-min observation window) increased significantly from 1 to 2 d in culture (Fig. 1*E*). The spread of synchronized signals from cell to cell was only rarely observed (Supplemental Video S1).

### Calcium stores in the endoplasmic reticulum are the main source of calcium transients in MM cells

To determine the source of the calcium transients, we applied 30 µM CPA, an inhibitor of the sarco-endoplasmic reticulum Ca^2+^-ATPase (SERCA pump), to deplete the calcium stores in the endoplasmic reticulum (ER) (Fig. 2*A*, *B*). Inhibition of the SERCA pump resulted, as expected, in a long and transient increase in cytosolic calcium. This was followed by an almost complete loss of calcium transients, indicating that the spontaneous calcium activity was dependent on calcium release from the ER. Furthermore, we found that influx of extracellular calcium does not appear to be necessary for the generation of cytosolic calcium transients. Exposure of kidneys to calcium-free solution containing 250 µM EGTA (Fig. 2*C*), 50 µM nifedipine (an inhibitor of voltage-gated calcium channels) (Fig. 2*D*), or 10 µM BTP-2 (a blocker of store-operated calcium entry) (Fig. 2*E*), had little or no effect on spontaneous calcium activity.

### Branching morphogenesis and nephron formation depends on calcium release from ER

**Figure 3:**
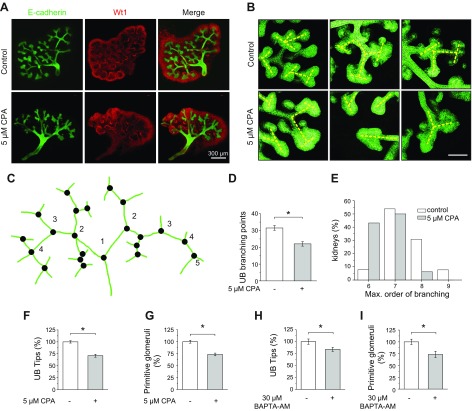
Chronic down-regulation of spontaneous calcium activity retarded the formation of new UB cells, primitive glomeruli, and tubule pattern formation. *A*) Whole-mount immunostaining of embryonic kidneys showing ureteric bud cells (E-cadherin, green) and mesenchymal cells (Wt1, red) in control and 5 µM CPA–treated kidneys. Scale bar, 300 µm. *B*) UB branching pattern in kidneys treated with 5 µM CPA for the last 24 h. UB tips have abnormal shape, and their number is decreased in comparison with control. The yellow dashed lines indicate an interpretation of the branching patterns. Most of the UB branches in control kidneys show a reiterative pattern of terminal bifurcation, with branches forming at right angles to their predecessors. Most of the UB branches in CPA-treated kidneys fail to conform to this pattern but display instead an abnormal shape and branching pattern. Scale bar, 50 µm. *C*) Measurement of UB branching points and order. Green lines are ureteric bud skeleton; black dots represent branching points of the ureteric bud tree. The skeleton was made by manual drawing on the fluorescent image of E-cadherin–labeled UB tree. Digits indicate order of branching. Total number of ureter branching and total number of branching with every order were determined. *D*) Chronic application of 5 µM CPA for 24 h reduced the number of UB branching points. Statistical significance (*P* < 0.05) was calculated by 2-tailed test, nonparametric *U* test(control group, *n* = 13; CPA-treated group, *n* = 15). *E*) Chronic application of 5 µM CPA for 24 h shifted the distribution of the maximal order of branching for the UB trees to lower numbers. *F*, *G*) Chronic application of 5 µM CPA for 24 h reduced the number of UB tips (*F*) and primitive glomeruli (*G*). Values of the control group were assigned as 100%. Statistical significance (*P* < 0.05) was calculated by ANOVA (control group, *n* = 13; CPA-treated group, *n* = 15). *H*, *I*) Incubation with the calcium chelator BAPTA-AM (30 µM) for 24 h reduced the number of UB tips (*H*) and primitive glomeruli (*I*). Values of the control group were assigned as 100%. Statistical significance was calculated by 2-tailed, nonparametric *U* test (*P* < 0.05; control group, *n* = 7; CPA-treated group, *n* = 8).

To assess the importance of spontaneous calcium activity in MM cells for ureter branching and nephron formation, the ER calcium stores were moderately depleted by a low-dose chronic application of CPA (5 µM) for 24 h from DIV1 to DIV2. Because CPA is a membrane-permeable drug, incubation for 24 h results in deep tissue penetration and more uniform distribution in the embryonic kidney than for the calcium indicator. CPA treatment resulted in a significant reduction of the number of calcium transients (Fig. 2*F*–*H*) and in suppression of branching morphogenesis (**Fig. 3**). Confocal imaging and morphologic analysis of immunofluorescence-stained kidneys showed that the UB tips appeared more swollen and irregular in CPA-treated than in control kidneys (Fig. 3*B*). The number of UB branching points and the UB branching order, assessed following the scheme introduced by Watanabe and Costantini (13), were significantly reduced in CPA-treated kidneys compared with control kidneys (Fig. 3*C*–*E*). The numbers of UB tips and primitive glomeruli in the CPA-treated kidneys were also significantly reduced (Fig. 3*F*, *G*).

**Figure 4:**
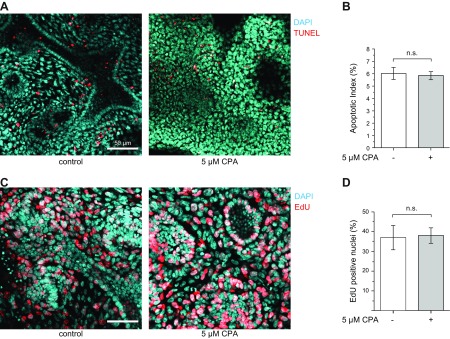
Calcium depletion from DIV1 to DIV2 has no effect on apoptosis and proliferation. *A*) Optical section (3 µm) images of whole-mount stained kidneys grown in control conditions and in the presence of 5 µM CPA. Red indicates apoptotic TUNEL-positive nuclei; cyan indicates DAPI nuclear counterstaining. Scale bar, 50 µm. *B*) Apoptotic index was calculated as the ratio of TUNEL-positive nuclei to the total number of DAPI stained nuclei. No difference was found between the control and the treated group. Statistical significance was calculated by 2-tailed, nonparametric *U* test (*n* = 4 in each group). N.s., not significant. *C*) Optical section (3 µm) images of whole-mount stained kidneys grown in control conditions and in the presence of 5 µM CPA from DIV1 to DIV2. Red, EdU-positive labeled nuclei; cyan, DAPI nuclear counterstaining. Scale bar, 50 µm. *D*) The proliferative index was calculated as the ratio of EdU-positive nuclei to the total number of nuclei counterstained with DAPI. No difference was found between control and treated groups. Statistical significance was calculated by 2-tailed, nonparametric *U* test (*n* = 5 in each group).

As an alternative approach to achieve down-regulation of the calcium activity, we applied the calcium chelator BAPTA-AM for 24 h from DIV1 to DIV2. This substance has the same membrane permeability characteristics as the calcium indicators used and only enters the first cell layer of the kidney, resulting in a restricted and localized down-regulation of calcium activity. The formation of UB tips and primitive glomeruli was reduced to a similar extent as in CPA-treated kidneys (Fig. 3*H*, *I*). Finally, a well-controlled balance between proliferation and apoptosis is a hallmark of embryonic pattern formation. These parameters were not affected by CPA treatment (**Fig. 4**).

### Deletion of the calcium sensor protein synaptotagmin 1 appears to have no effect on ureter budding and branching

Most secretory processes are regulated by calcium signaling pathways that act on the calcium-dependent synaptotagmin protein family (14). Synaptotagmin 1 (Syt1) is the most well-studied synaptotagmin prototype. Syt1 RNA is, according to Gudmap, expressed in the rodent kidney throughout embryogenesis (15, 16). To test whether release of paracrine factors that initiate the interaction between MM and UB cells is synaptotagmin dependent, we determined ureter buds and branches in E14 embryos from Syt1 knockout mouse. We found no quantitative or morphologic differences between wild-type, heterozygote, and homozygote embryos. The number of Syt1^−/−^ embryos detectable at E14 was lower than expected according to the Mendelian law of inheritance (44^+/+^, 43^+/−^, 6^−/−^). Because we cannot exclude that the few surviving double-negative embryos represent a population that has undergone positive selection, we do not consider these results conclusive.

## DISCUSSION

Our study has identified calcium as a factor that is important for the control of morphogenesis in the kidney. Branching morphogenesis has been described as a self-organized process with genetically programmed and stochastic components. We suggest that calcium transients are one of the factors that contribute to the stochastic components of branching morphogenesis.

The role of calcium signaling for embryogenesis and organogenesis of early vertebrates is well documented. For example, spontaneous calcium activity has been observed after egg fertilization (17–19), during early development of zebra fish (20) and frog (21, 22), in stem cells (23, 24), and in developing neurons (25, 26).

The spontaneous calcium activity we observed in MM cells appeared random, and we could not identify spatial or temporal correlation within or between cells. The shape and duration of calcium transients was also highly irregular and differed markedly from the calcium waves that are typical for activation of Gq coupled receptors and release of inositol trisphosphate.

On the basis of the irregular characteristics of the calcium signals, we propose that mechano-transduction from the external environment to intracellular stores is a possible trigger mechanism for calcium signals (27). Mechanical stimulation has been found to trigger calcium release in human mesangial stem cells (28). In our study, we recorded calcium signals from the proliferating mesenchymal tissue, an environment that, during organogenesis, creates and is exposed to mechanical forces from surrounding proliferating cells.

Our study indicates that spontaneous calcium activity in MM cells plays an important role in kidney development. Quantification of UB tips and branching order shows that the complexity of the kidney is reduced when the frequency of calcium transients is reduced. Nephron development depends on secretion of paracrine factors, such as fibroblast growth factor and WNT9B, which are produced in UB cells and act on MM cells to stimulate mesenchymal-epithelial transition (29–32). The embryonic kidney continues to grow after a reduction of calcium signals, but with a lower probability for branching. Our data are in line with and support theoretical studies demonstrating that branching morphogenesis can be described as a stochastic and self-organizing process (4). Release of diffusible morphogenic factors at the UB-MM interface could modify and control the stochastic process that drives branching morphogenesis and lead to a self-organized morphology of the kidney (5–7).

We propose that the spontaneous calcium signals observed in MM cells are, along with stochastic components, among the factors that contribute to the process that drives branching morphogenesis. Blockade of the calcium signal reduces the probability of branching but does not eliminate the drive for branching morphogenesis.

The identification of spontaneous calcium activity in MM cells and their functional importance has clinical implications. The development of bioengineering methods for kidney generation is a rapidly expanding field and might require the reconstitution of the interaction between MM and UB cells (33). The calcium activity in MM cells is a good candidate for a biomarker of MM health and integrity. Because low nephron endowment has an adverse effect on the outcome of many kidney diseases, it will be important to study to which disturbances in maternal calcium homeostasis and/or drugs that target calcium transporters influence the fetal kidney.

## Supplementary Material

This article includes supplemental data. Please visit *http://www.fasebj.org* to obtain this information.

## Abbreviations

AM: acetoxymethyl
CPA: cyclopiazonic acid
DIV: days in vitro
ER: endoplasmic reticulum
MM: metanephric mesenchyme
NA: numerical aperture
PBST: PBS containing 0.1% Triton X-100
Syt1: synaptotagmin 1
UB: ureteric bud
