# Supplementary material for: Spontaneous calcium activity in metanephric mesenchymal cells regulates branching morphogenesis in the embryonic kidney
Source: FASEB J. 2018 Nov 29;33(3):4089–96. doi: 10.1096/fj.201802054R (PMC6404591; doi:10.1096/fj.201802054R)
Supplement: Supplementary file 1 [file fj.201802054R.sd1.docx]

## Supplemental material

**Video 1**. Calcium recording (40 minutes) of a DIV2 embryonic kidney loaded with Oregon Green Bapta-1 AM. (Duration of the compressed video is 30 s, 40 frames per second).
